# Association of triglyceride glucose index with cardiovascular autonomic neuropathy in type 2 diabetes mellitus

**DOI:** 10.3389/fcvm.2026.1829752

**Published:** 2026-07-09

**Authors:** Feijuan Kong, Ziqi Wang, Qiyu Yang, Jingzhu Wu, Shanshan Tang, Mingyu Gu, Xiaoying Ding, Yufan Wang

**Affiliations:** 1 Department of Endocrinology and Metabolism, Shanghai General Hospital Affiliated to Medical College of Shanghai Jiao Tong University, Shanghai, China; 2 School of Medicine, University of Electronic Science and Technology of China, Chengdu, China

**Keywords:** cardiovascular autonomic neuropathy, diabetic complications, insulin resistance, triglyceride glucose index, type 2 diabetes mellitus

## Abstract

**Background:**

Cardiovascular autonomic neuropathy (CAN), defined as impaired autonomic control of the cardiovascular system, is a highly prevalent yet frequently underdiagnosed microvascular complication of diabetes. The triglyceride-glucose (TyG) index, a well-validated surrogate marker of insulin resistance, may have considerable clinical relevance for CAN. Nevertheless, the association between the TyG index and CAN in patients with type 2 diabetes mellitus (T2DM) remains unclear.

**Methods:**

A total of 230 hospitalized patients with T2DM were recruited from the Department of Endocrinology and Metabolism, Shanghai General Hospital. CAN status was evaluated using the cardiovascular autonomic reflex test (CART) and heart rate variability (HRV) measurements. Meanwhile, demographic characteristics and relevant clinical data were collected, and the TyG index was calculated based on fasting blood glucose and triglyceride levels for subsequent statistical analysis.

**Results:**

The prevalence of CAN in our cohort of 230 patients was 61.3% (141 cases). Patients with CAN exhibited a significantly higher TyG index compared to those without CAN. The TyG index demonstrated positive correlations with CART parameters (E:I difference, *r* = 0.301; Valsalva ratio, *r* = 0.362; 30:15 ratio, *r* = 0.166; BP change, *r* = 0.229; all *P* < 0.05) and inverse correlations with HRV indices (SDNN, *r* = −0.257; RMSSD, *r* = −0.226; pNN50, *r* = −0.226; VLF, *r* = −0.221; LF, *r* = −0.253; HF, *r* = −0.212; TP, *r* = −0.249; all *P* < 0.01). Moreover, after adjusting for demographic and metabolic confounders, the TyG index remained a significant independent predictor of CAN. ROC curve analysis indicated that the TyG index possesses favorable discriminatory power for CAN, with an AUC of 0.854 (95% CI: 0.796–0.911, *P* < 0.001).

**Conclusions:**

The TyG index shows a distinct association with CAN assessment parameters. As a well-established, effective, and practical surrogate marker of IR, this finding highlights the clinical relevance of the TyG index in diabetes-related complications.

## 1 Introduction

Diabetes mellitus (DM) has emerged as one of the fastest-growing noncommunicable diseases worldwide (1). As reported in the 11th edition of the International Diabetes Federation (IDF) Global Diabetes Map, there were 589 million adults aged 20–79 years living with diabetes in 2024, representing 11.1% of the global population in this age range. This proportion is projected to increase to 13% by 2050, with the total number surpassing 853 million (2). The latest large-scale epidemiological survey in China revealed that the age-standardized prevalence of diabetes in adults aged 18 and above has reached 12.4%, equating to approximately 130 million affected individuals. Moreover, coupled with a prediabetes (characterized by impaired glucose tolerance and/or impaired fasting glucose) prevalence of 38.1%, the above data indicate that over half (50.5%) of the adult population currently live with dysglycemia (3). DM, a highly prevalent chronic disease, triggers both macrovascular and microvascular damage through diverse pathological mechanisms such as oxidative stress, accumulation of advanced glycation end products (AGEs), activation of protein kinase C (PKC), and upregulation of the polyol pathway (1). These processes ultimately cause a spectrum of diabetic complications that impair patients' quality of life and overall survival.

Cardiovascular autonomic neuropathy (CAN) is a significant yet frequently overlooked microvascular complication of diabetes, largely due to its asymptomatic nature in early stages (4, 5). CAN prevalence in patients with T2DM ranges from 12% to 73% and correlates positively with disease duration (6, 7). Given the global increase in T2DM prevalence, the incidence of CAN is projected to rise substantially in the coming decades (7). Strong evidence establishes CAN as a significant independent predictor of adverse cardiovascular events in DM (4, 7, 8). A recent meta-analysis demonstrated that DM individuals with CAN face a threefold higher risk of future cardiovascular morbidity and mortality compared with those without CAN (8). Notably, recent studies have highlighted the occurrence of CAN even in the prediabetic stage, suggesting early involvement of the autonomic nervous system (9, 10). Nevertheless, CAN remains persistently underrecognized in clinical practice. Early detection of CAN is critical for halting disease progression and mitigating associated cardiovascular risks. The current diagnostic paradigm relies on two principal approaches: the cardiovascular autonomic reflex test (CART), which remains the gold standard, and the analysis of heart rate variability (HRV) indices. The latter has emerged as a sensitive and clinically practical tool for point-of-care assessment (4, 5).

The pathogenesis and progression of CAN in diabetes are driven by complex and multifactorial mechanisms that are incompletely understood. Accumulating evidence implicates several key pathways, including the accumulation of AGEs, activation of the hexosamine, PKC, and polyol pathways—leading to subsequent osmotic and oxidative stress—as well as neuronal ischemia resulting from diabetic microangiopathy and disturbed lipid metabolism (4, 11–13). Furthermore, clinical conditions, e.g., insulin resistance (IR), elevated body mass index (BMI), increased waist circumference, hypertriglyceridemia, and hypertension, are consistently associated with abnormalities in autonomic function indices (14). Of note, the triglyceride-glucose (TyG) index has been increasingly recognized as a simple and effective surrogate marker of systemic metabolic dysregulation. Functioning as a metabolic alarm, the TyG index is derived from the product of fasting plasma glucose (FPG) and triglyceride (TG) levels, demonstrating a strong correlation with the severity of IR. Beyond its established associations with T2DM and diabetic nephropathy (DN), the TyG index serves as a pivotal biomarker, linking IR to a broad spectrum of cardio-cerebrovascular and metabolic complications (15–17). However, current literature has a paucity of evidence establishing a direct relationship between the TyG index and CAN in patients with T2DM.

Detecting the TyG index associated with CAN would enhance our understanding of disease pathogenesis. This advancement holds the potential to facilitate the development of diagnostic or prognostic markers suitable for point-of-care testing and to inform novel targeted therapeutic strategies. Thus, the primary objective of this study was to investigate the associations of the TyG index with CAN parameters in individuals with T2DM.

## 2 Methods

### 2.1 Study population

**Figure 1:**
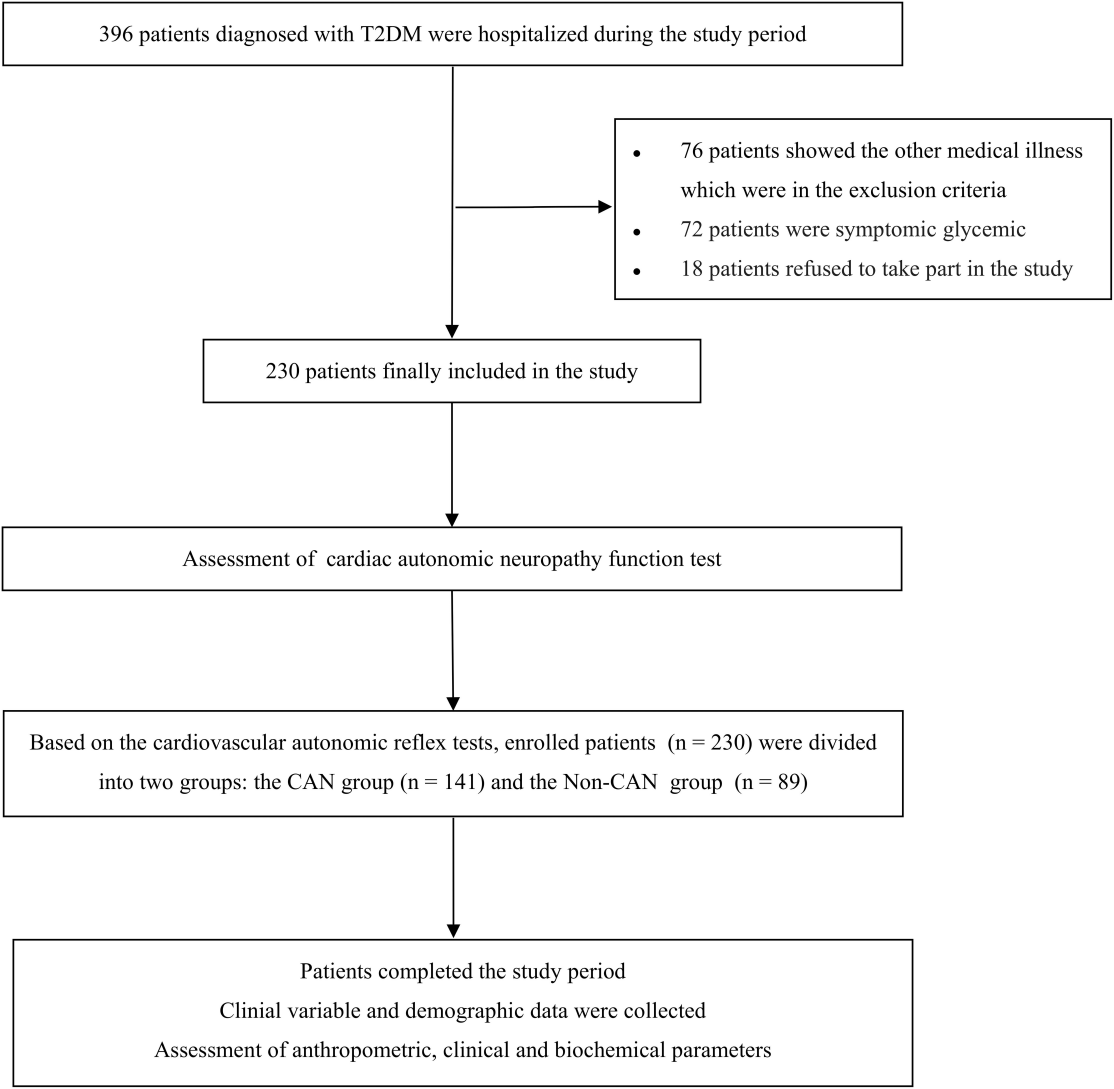
Flow chart showing the study participants. T2DM, Type 2 diabetes mellitus; CAN, Cardiac autonomic neuropathy.

Between January 1 and December 31, 2025, a total of 396 hospitalized patients with T2DM were screened and underwent the cardiovascular autonomic reflex test (CART) in the Department of Endocrinology and Metabolism at Shanghai General Hospital. Subsequent exclusion of 76 patients with comorbid medical conditions meeting the exclusion criteria, 72 with symptomatic hyperglycemia, and 18 who refused participation resulted in a final cohort of 230 patients for the cross-sectional analysis (Figure 1). Depending on CART results, the cohort was divided into two groups: the CAN (141 T2DM patients with CAN) and non-CAN (89 T2DM patients without evidence of CAN) groups. Eligible participants were required to be at least 18 years of age, have a confirmed diagnosis of T2DM per WHO or ADA criteria, be conscious and lucid, and possess the cognitive capacity to understand and cooperate during the CART. Exclusion criteria were: type 1, gestational, or secondary diabetes; acute diabetic complications (e.g., ketoacidosis, hyperosmolar state); use of a pacemaker or antiarrhythmic drugs; a history of major neurological or cardiac disease; anemia or severe hepatic, renal, or other systemic diseases; indication of proliferative retinopathy or retinal hemorrhage; use of medications that interfere with autonomic function (including beta-blockers, antihistamines, antitussives, antidepressants, or diuretics); refusal to participate in the study.

Ethical approval was obtained from the Institutional Review Board of Shanghai General Hospital (IRB No. 2025−682), and the requirement for informed consent was waived. The study was conducted in full accordance with all applicable guidelines and regulations.

### 2.2 Data collection

The clinicodemographic data collected for analysis included age, sex, duration of DM, body mass index (BMI), smoking status, alcohol consumption, and previous history of hypertension, cardiovascular disease, diabetic retinopathy (DR), and diabetic peripheral neuropathy (DPN). DM duration was determined from the date of initial diagnosis to the date of the CART. Participants were categorized as smokers if they were current or former smokers. Hypertension was defined as systolic blood pressure ≥140 mmHg, diastolic blood pressure ≥90 mmHg, or current use of antihypertensive medications. Cardiovascular disease was defined as a documented history of myocardial infarction, peripheral arterial occlusive disease, or cerebrovascular disease. DPN was assessed with the Toronto Clinical Scoring System (TCSS) or the Michigan Neuropathy Screening Instrument (MNSI). A diagnosis of DR was established based on a comprehensive clinical examination by ophthalmologists.

The laboratory parameters assessed comprised glycated hemoglobin (HbA1c), glycated albumin (GA), fasting blood glucose (FBG), aspartate aminotransferase (AST), alanine aminotransferase (ALT), estimated glomerular filtration rate (eGFR), urinary albumin-to-creatinine ratio (uACR), uric acid, total cholesterol (TC), triglycerides (TG), high-density lipoprotein cholesterol (HDL-C), and low-density lipoprotein cholesterol (LDL-C).

The triglyceride-glucose (TyG) index was calculated using the following formula: Ln [FBG (mg/dL) × TG (mg/dL)/2].

### 2.3 Assessment of CAN

CAN evaluations in this cohort were conducted under standardized conditions with a multi-parameter cardiovascular autonomic function testing system (Model R6000; Ruimeng Medical Technology Co., Ltd., Shenzhen, China). Briefly, participants were instructed to refrain from strenuous exercise on both the day before and the day of the test. Within the 12 h preceding the examination, smoking and consumption of coffee, strong tea, and alcohol were prohibited. Additionally, antihypertensive medications were withheld during this period.

Following confirmation of eligibility, participants rested quietly in the supine position for a minimum of 20 min. Subsequently, a 5-minute baseline recording of resting heart rate variability (HRV) was acquired. Cardiac autonomic function was evaluated by HRV parameters, considering both the time and frequency domains. Time-domain indices comprised the standard deviation of normal-to-normal (NN) intervals (SDNN), the root mean square of successive differences between adjacent NN intervals (RMSSD), and the percentage of adjacent NN intervals differing by more than 50 ms (pNN50). Frequency-domain indices, obtained through spectral analysis, included total power (TP), very low-frequency power (VLF, 0.003-0.04 Hz), low-frequency power (LF, 0.04-0.15 Hz), high-frequency power (HF, 0.15-0.40 Hz), and the LF/HF ratio.

CAN diagnosis was subsequently confirmed in all participants through administration of criterion-standard CART, consisting of four core autonomic function assessments: heart rate response to deep breathing (E:I difference), heart rate response to standing (30:15 ratio), heart rate response to the Valsalva maneuver (Valsalva ratio), and blood pressure response to posture change (BP change). Of the 4 tests, the first three primarily assess parasympathetic function, while the last evaluates sympathetic activity; all are characterized by high sensitivity, specificity, and reproducibility.

The heart rate response to deep breathing (E:I difference) was defined as the median difference between the maximum and minimum heart rates over six complete respiratory cycles during one minute. Values were categorized as normal (≥ 15 beats), borderline (11–14 beats), or abnormal (≤ 10 beats). The heart rate response to postural change (from lying to standing) was quantified by the 30:15 ratio, i.e., the ratio of the longest R-R interval around the 30th beat to the shortest R-R interval around the 15th beat on the electrocardiogram after standing. Based on this ratio, responses were classified as normal (≥ 1.04), borderline (1.01–1.03), or abnormal (≤ 1.00). The Valsalva ratio, defined as the ratio of the longest R-R interval after the maneuver to the shortest R-R interval during the maneuver, was employed to quantify the heart rate response to the Valsalva maneuver. Participants were then categorized as having a normal (≥ 1.21), borderline (1.11–1.20), or abnormal (≤ 1.10) response based on this ratio. The blood pressure response to posture change (BP differences) was assessed. After measuring supine blood pressure, a second measurement was obtained within 2 min of active standing. The test outcome was defined as the difference in systolic pressure (standing minus supine). This orthostatic systolic change was categorized as normal (≤ 10 mmHg decrease), borderline (11–29 mmHg decrease), or abnormal (≥ 30 mmHg decrease). The results of the four CARTs were scored as follows: normal = 0 point, borderline = 0.5 point, and abnormal = 1 point. The total score was then used to classify participants as having no CAN (score: 0–0.5 point) or CAN (≥ 1 point), with CAN further subclassified as early CAN (score: 1–1.5 points), definite CAN (score: 2–3 points) or severe CAN (score: ≥ 3.5 points).

### 2.4 Statistical analysis

Clinical characteristics are presented as frequency (percentage) for categorical variables, and as mean ± standard deviation (SD) or median (first–third quartiles; min–max) for continuous variables, depending on their distribution. Group comparisons were performed by the chi-square test for categorical variables. For continuous variables, the independent Student's t-test was applied for normally distributed data, and the Mann–Whitney U test for non-normally distributed data. Correlations between clinical parameters and CAN measures were evaluated using the Spearman's rank correlation coefficient. To examine potential independent associations, two regression models were performed: a multivariable linear regression to assess the relationships between the TyG index and HRV parameters, and a multivariable logistic regression to identify independent factors contributing to CAN diagnosis. Receiver operating characteristic (ROC) curve analysis was adopted to assess the diagnostic efficacy of the TyG index for CAN. The area under the curve (AUC) was calculated to reflect overall diagnostic accuracy. The optimal cutoff value was determined by maximizing the Youden index, and the corresponding sensitivity and specificity were obtained. A two-sided P-value < 0.05 was considered statistically significant. All analyses were conducted with SPSS Statistics version 27 (IBM Corp., Armonk, NY, USA).

## 3 Results

### 3.1 Characteristics of participants

A total of 230 participants were included in the final analysis. Their clinical characteristics stratified by CAN status are presented in Table 1. The mean participant age in the cohort was 56.56 ± 0.98 years, with a male-to-female ratio of 1.98:1. Based on CART results, 89 cases (38.70%) were classified as non-CAN and 141 (61.30%) as CAN. No statistically significant differences were observed in demographic, metabolic, or other clinical characteristics between the two groups, except for age, diabetes duration, eGFR, FBG, and TG. Patients in the CAN group were significantly older and had longer diabetes duration, lower eGFR, higher FBG and TG levels, and higher prevalence rates of cardiovascular disease and DPN compared with the non-CAN group (*P* < 0.05). In addition, no significant difference was observed between the two groups in the use of insulin, GLP-1RAs, SGLT2is, finerenone, angiotensin-converting enzyme inhibitors (ACEIs)/angiotensin receptor blockers (ARBs), or statins. Of note, the TyG index was significantly elevated in the CAN group relative to the non-CAN group.

**Table 1:** Baseline characteristics in the CAN and Non-CAN groups.

Characteristic	Total	Non-CAN group	CAN group	*P* value
**N (%)**	230	89 (38.70)	141 (61.30)	
**Gender, *n* (%)**				0.405
Male	133 (57.83)	45 (33.83)	88 (66.17)	
Female	97 (42.17)	44 (45.36)	53 (54.64)	
**Age, *n* (%)**				0.036
< 50 years	53 (23.04)	26 (49.06)	27 (50.94)	
50–59 years	61 (26.52)	22 (36.07)	39 (63.93)	
60–69 years	93 (40.43)	32 (34.41)	61 (65.59)	
≥ 70 years	23 (10.01)	10 (43.48)	13 (56.52)	
**DM duration, *n* (%)**				0.049
< 5 years	74 (36.36)	32 (43.24)	42 (56.76)	
5–10 years	81 (34.66)	29 (35.80)	52 (64.20)	
11–14 years	32 (6.82)	13 (40.63)	19 (59.37)	
≥ 15 years	43 (22.16)	16 (37.21)	27 (62.79)	
**BMI, *n* (%)**				0.665
< 18.5 kg/m^2^	8 (3.48)	4 (50.00)	4 (50.00)	
18.5–23.9 kg/m^2^	92 (40.00)	36 (39.13)	56 (60.87)	
24–27.9 kg/m^2^	92 (40.00)	33 (35.87)	59 (64.13)	
≥ 28 kg/m^2^	38 (16.52)	17 (44.74)	21 (55.26)	
**eGFR, *n* (%)**				0.011
< 60 mL/min/1.73 m^2^	41 (17.83)	17 (41.46)	24 (58.54)	
≥ 60 mL/min/1.73 m^2^	189 (82.17)	73 (38.62)	116 (61.38)	
**uACR, *n* (%)**				0.115
< 30 µg/mg	162 (70.43)	65 (40.12)	97 (59.88)	
≥ 30 µg/mg	68 (29.57)	25 (36.76)	43 (63.24)	
**HbA1c, *n* (%)**				0.393
< 7%	78 (33.91)	32 (41.03)	46 (58.97)	
7–8.9%	74 (32.18)	27 (36.49)	47 (63.51)	
≥ 9%	78 (33.91)	31 (39.74)	47 (60.26)	
**GA, %**	20.28 ± 0.47	20.17 ± 0.81	20.35 ± 0.57	0.528
**FBG, mmol/L**	7.08 (5.66, 9.00)	5.70 (4.83, 7.80)	7.81 (6.36, 9.40)	0.001
**TG, mmol/L**	1.86 (1,38, 5.93)	1.35 (0.96, 1.60)	2.15 (1.80, 3.11)	0.001
**TyG index**	9.27 ± 0.05	8.74 ± 0.07	9.7 ± 0.05	0.002
**TC, mmol/L**	4.38 ± 0.12	4.32 ± 0.15	4.41 ± 0.16	0.832
**HDL-C, mmol/L**	1.12 ± 0.34	1.12 ± 0.36	1.12 ± 0.27	0.404
**LDL-C, mmol/L**	2.72 ± 0.07	2.75 ± 0.11	2.70 ± 0.09	0.576
**AST, U/L**	27.20 ± 1.53	28.88 ± 3.25	26.26 ± 1.54	0.526
**ALT, U/L**	32.35 ± 2.50	35.04 ± 5.28	30.85 ± 2.56	0.486
**UA,** µ**mol/L**	340.47 ± 18.03	326.30 ± 10.75	348.36 ± 27.46	0.630
**Hypertension, *n* (%)**	79 (44.89)	28 (44.44)	51 (45.13)	0.930
**Cardiovascular disease, *n* (%)**	147 (83.52)	47 (74.60)	100 (88.50)	0.021
**Smoking, *n* (%)**	29 (16.48)	9 (14.29)	20 (17.70)	0.558
**Drinking habits, *n* (%)**	19 (10.80)	4 (63.49)	15 (13.27)	0.156
**DR, *n* (%)**	30 (17.05)	8 (12.70)	22 (19.47)	0.252
**DPN, *n* (%)**	84 (47.73)	29 (46.03)	75 (66.37)	0.009
**Insulin, *n* (%)**	111 (63.07)	39 (61.90)	72 (63.72)	0.811
**ACEi/ARB, *n* (%)**	76 (43.18)	28 (44.44)	48 (42.48)	0.801
**SGLT2i, *n* (%)**	133 (75.57)	42 (66.67)	91 (80.53)	0.040
**GLP-1RA, *n* (%)**	128 (72.73)	44 (69.84)	84 (74.34)	0.521
**Finerenone, *n* (%)**	32 (18.18)	9 (14.29)	23 (20.35)	0.317
**Statin, *n* (%)**	156 (88.64)	53 (84.15)	103 (91.15)	0.159

CAN, cardiac autonomic neuropathy; BMI, body mass index; DM, diabetes mellitus; eGFR, estimated glomerular filtration rate; uACR, urinary albumin-creatinine ratio; HbA1c, glycated hemoglobin; GA, glycated albumin; FBG, fasting blood glucose; TC, total cholesterol; TG, triglycerides; HDL-C, high-density lipoprotein cholesterol; LDL-C, low-density lipoprotein cholesterol; TyG index, triglyceride-glucose index; AST, aspartate aminotransferase; ALT, alanine aminotransferase; DR, diabetic retinopathy; DPN, diabetic peripheral neuropathy; ACEi, angiotensin converting enzyme inhibitors; ARB, angiotensin II receptor blockers; SGLT2i, sodium–glucose cotransporter-2 inhibitors; GLP-1RA, glucagon-like peptide-1 receptor agonists.

### 3.2 Comparison of CART scores

CART outcomes were defined by four key measures: E:I difference, Valsalva ratio, 30:15 ratio, and BP response to postural change. The results were scored as follows: normal = 0 point, borderline = 0.5 point, and abnormal = 1 point. Compared with the non-CAN group, the CAN group demonstrated significant differences in E:I difference, Valsalva ratio, 30:15 ratio, and BP difference after postural change (*P* < 0.001, Table 2).

**Table 2:** CART index scores in the CAN and Non-CAN groups.

Group	E:I difference			Valsalva ratio			30:15 ratio			BP differences after postural change		
	0	0.5	1	0	0.5	1	0	0.5	1	0	0.5	1
DCAN	55	34	52	46	39	56	68	46	27	61	66	14
Non-DCAN	66	23	0	61	28	0	72	17	0	70	19	0
*P* value	< 0.001			< 0.001			< 0.001			< 0.001		

CAN, cardiac autonomic neuropathy; CART, cardiac autonomic reflex tests; E/I difference, heart rate response to deep breathing; 30/15 ratio, heart rate response to standing; BP, blood pressure.

### 3.3 Comparison of HRV indices

HRV analysis covered both time-domain (SDNN, RMSSD, and pNN50) and frequency-domain (VLF, LF, HF, LF/HF ratio, and TP) indices. Significant differences were detected in multiple HRV indices between the two groups. Time-domain (SDNN, RMSSD, and pNN50) and frequency-domain (VLF, LF, HF, and TP) indexes all demonstrated statistically significant differences (*P* < 0.05, Table 3).

**Table 3:** HRV indexes in the CAN and non-CAN groups.

Index	Non-CAN	CAN	*P* value
SDNN (ms)	28.25 ± 1.27	22.12 ± 1.01	< 0.001
RMSSD (ms)	20.70 ± 1.29	16.18 ± 0.96	0.005
pNN50 (%)	3.46 ± 0.63	2.40 ± 0.52	< 0.001
VLF (ms^2^)	133.30 ± 13.89	98.16 ± 10.16	0.042
LF (ms^2^)	444.10 ± 46.65	344.90 ± 58.30	< 0.001
HF (ms^2^)	138.00 ± 18.32	97.15 ± 15.94	< 0.001
LF/HF	4.63 ± 0.47	4.94 ± 0.32	0.147
TP (ms^2^)	715.40 ± 66.95	540.20 ± 72.39	< 0.001

CAN, cardiac autonomic neuropathy; HRV, heart rate variability; SDNN, standard deviation of NN intervals; RMSSD, root mean square of successive differences between adjacent N-N intervals; pNN50, percentage of adjacent NN intervals differing by more than 50 ms; VLF, very low-frequency; LF, low-frequency; HF, high-frequency; TP, total power.

### 3.4 Associations of CAN severity with HRV measures

Based on CART scores, 230 patients with T2DM were categorized into four groups: non-CAN (0–0.5 point; n = 89, 38.70%), early CAN (1–1.5 points; *n* = 114, 49.57%), definite CAN (2–3 points; *n* = 25, 10.87%), and severe CAN (≥ 3.5 points; *n* = 2, 0.86%). Correlation analysis between CAN severity and HRV indices showed that CAN severity was negatively correlated with SDNN (*r* = –0.329, 95% CI: −0.458 to −0.186), RMSSD (*r* = –0.296, 95% CI: −0.429 to −0.150), pNN50 (*r* = –0.290, 95% CI: −0.423 to −0.144), VLF (*r* = –0.233, 95% CI: −0.372 to −0.084), LF (*r* = –0.296, 95% CI: −0.429 to −0.150), HF (*r* = –0.303, 95% CI: −0.435 to −0.158), and TP (*r* = –0.301, 95% CI: −0.434 to −0.156) (*P* < 0.001).

### 3.5 TyG index and CAN measures

**Figure 2:**
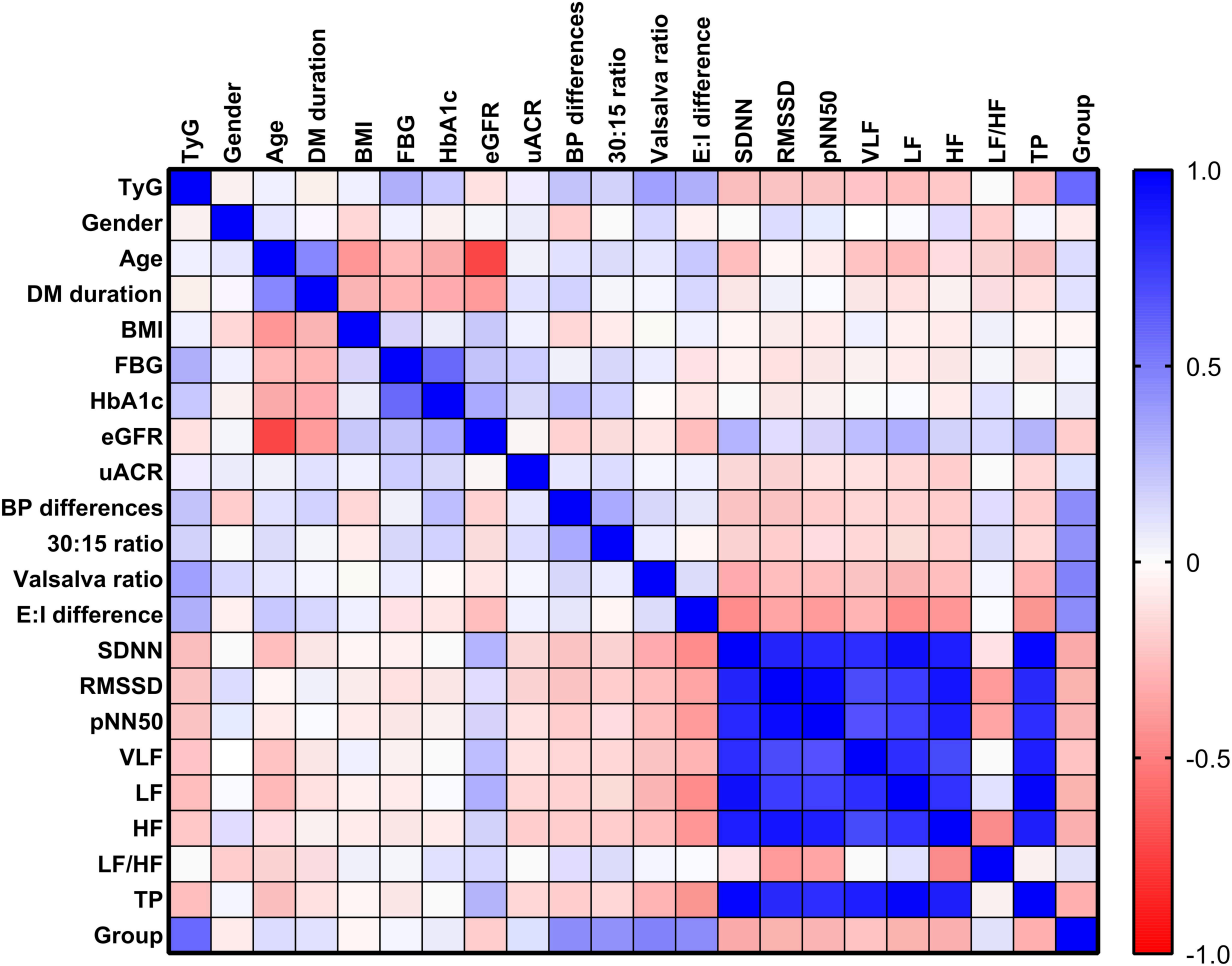
Baseline parameters correlations with CAN measures in T2DM cases. CAN, Cardiac autonomic neuropathy; BMI, Body mass index; T2DM, Type 2 diabetes mellitus; eGFR, Estimated glomerular filtration rate; uACR, Urinary albumin-creatinine ratio; HbA1c, Glycated hemoglobin; FBG, fasting blood glucose; TyG index, Triglyceride-glucose index; E/I difference, Heart rate response to deep breathing; 30/15 ratio, Heart rate response to standing; BP, Blood pressure; SDNN, Standard deviation of NN intervals; RMSSD, Root mean square of successive differences between adjacent N-N intervals; pNN50, Percentage of adjacent NN intervals differing by more than 50 ms; VLF, Very low-frequency; LF, Low-frequency; HF: High-frequency; TP: Total power.

Figure 2 presents the Spearman's rank correlation coefficients between baseline parameters and CAN measures in T2DM cases.

As shown in Figure 2, SDNN, VLF, LF, and TP were inversely correlated with age (*r* = −0.244, 95% CI: −0.382 to −0.096, *P* = 0.001; *r* = −0.234, 95% CI: −0.373 to −0.085, *P* = 0.002; *r* = −0.267, 95% CI: −0.403 to −0.120, *P* < 0.001; and *r* = −0.240, 95% CI: −0.378 to −0.091, *P* = 0.001; respectively) and positively correlated with eGFR (*r* = 0.293, 95% CI: 0.147–0.426, *P* < 0.001; *r* = 0.247, 95% CI: 0.099–0.385, *P* = 0.001; *r* = 0.304, 95% CI: 0.159–0.436, *P* = 0.028; *r* = 0.285, 95% CI: 0.138–0.419, *P* < 0.001; respectively). SDNN, RMSSD, LF, HF, and TP were negatively associated with uACR (*r* = −0.148, 95% CI: −0.294 to −0.004, *P* = 0.046; *r* = −0.162, 95% CI: −0.307 to −0.010, *P* = 0.032; *r* = −0.155, 95% CI: −0.230 to −0.003, *P* = 0.040; *r* = −0.177, 95% CI: −0.321 to −0.026, *P* = 0.018; and *r* = −0.153, 95% CI: −0.299 to −0.001, *P* = 0.042; respectively). Furthermore, the presence of CAN was significantly associated with eGFR (*r* = −0.192, 95% CI: −0.333 to −0.040, *P* = 0.011).

Of note, among the comprehensive panel of indices examined, the TyG index showed notably significant inverse associations with several HRV indices, including SDNN (*r* = −0.257, 95% CI: −0.394 to −0.109, *P* < 0.001), RMSSD (*r* = −0.226, 95% CI: −0.366 to −0.076, *P* = 0.003), pNN50 (*r* = −0.226, 95% CI: −0.366 to −0.076, *P* = 0.003), VLF (*r* = −0.221, 95% CI: −0.361 to −0.071, *P* = 0.003), LF (*r* = −0.253, 95% CI: −0.390 to −0.105, *P* < 0.001), HF (*r* = −0.212, 95% CI: −0.353 to −0.062, *P* = 0.004), and TP (*r* = −0.249, 95% CI: −0.386 to −0.100, *P* < 0.001). Meanwhile, the TyG index was positively correlated with multiple CART parameters, including E:I difference (*r* = 0.301, 95% CI: 0.156–0.434, *P* < 0.001), Valsalva ratio (*r* = 0.362, 95% CI: 0.223–0.488, *P* < 0.001), 30:15 ratio (*r* = 0.166, 95% CI: 0.014–0.310, *P* = 0.027), and BP change (*r* = 0.229, 0.079–0.368, *P* = 0.002). Moreover, Spearman correlation analysis showed a significant positive correlation between TyG index and CAN severity (*r* = 0.554, 95% CI: 0.439–0.652, *P* < 0.001).

Given that the TyG index demonstrated the strongest and most consistent associations with CAN measures, subsequent analyses focused on this index.

### 3.6 Validation of the association between TyG index and CAN

**Figure 3:**
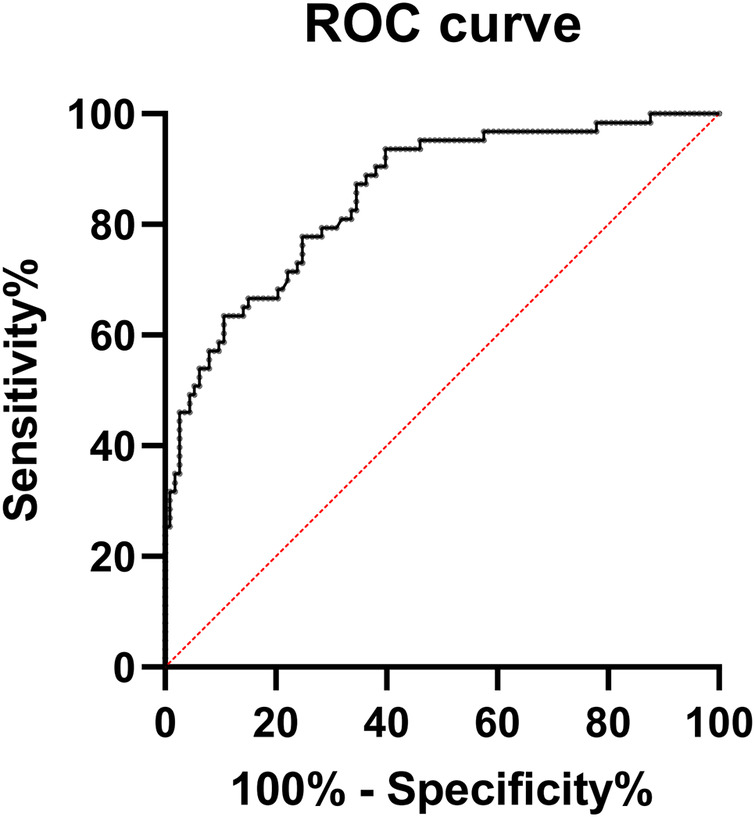
ROC curve analysis of the TyG index for predicting CAN. TyG index,Triglyceride-glucose index; CAN, Cardiac autonomic neuropathy.

In patients with CAN, correlation analysis revealed a very strong association between SDNN and RMSSD (*r* = 0.875, 95% CI: 0.822–0.913, *P* < 0.001), indicating marked sympathetic impairment. Furthermore, TP exhibited strong correlations with both LF (*r* = 0.971, 95% CI: 0.958–0.980, *P* < 0.001) and HF (*r* = 0.893, 95% CI: 0.846–0.926, *P* < 0.001), indicating concurrent involvement of sympathetic and parasympathetic pathways in cardiac autonomic injury. Subsequently, to investigate the potential of the TyG index as an independent predictor of cardiac autonomic dysfunction, a multivariable linear regression analysis was conducted. After adjustment for sex, age, diabetes duration, BMI, HbA1c, FPG, eGFR, and uACR in a linear regression model (Table 4), the TyG index emerged as the strongest independent predictor of SDNN, RMSSD, HF, and TP. Strikingly, even after adjustment for key demographic and metabolic confounders in a multivariable logistic regression model, the TyG index maintained an independent association with CAN (Table 5). Furthermore, ROC curve analysis indicated that the TyG index possesses favorable discriminatory power for CAN, with an AUC of 0.854 (95% CI: 0.796–0.911, *P* < 0.001) in Figure 3. The optimal cutoff value was determined as 9.16 based on the Youden index, with a sensitivity of 77.8% and specificity of 75.2%. Even after excluding patients with DPN/CVD, the TyG index still maintained strong diagnostic performance (AUC = 0.794, 95% CI: 0.700–0.888, *P* < 0.001; AUC = 0.813, 95% CI: 0.759–0.908, *P* < 0.001;respectively).

**Table 4:** Multiple linear regression analysis with HRV parameters as dependent variables.

Variable	SDNN			RMSSD			HF			TP		
	Coefficient *β*	95% CI	*P* value	Coefficient β	95% CI	*P* value	Coefficient β	95% CI	*P* value	Coefficient β	95% CI	*P* value
Gender	−0.880	−4.160 to 2.400	0.597	0.595	−0.686 to 1.877	0.339	0.121	−0.402 to 0.643	0.649	−0.678	−0.281 to 0.145	0.531
Age	−0.256	−0.421 to −0.090	0.003	−0.114	−0.279 to 0.052	0177	−0.589	−0.725 to −0.454	0.022	−0.188	−0.294 to −0.086	0.001
DM duration	0.095	−0.145 to 0.335	0.434	0.243	0.003 to 0.483	0.047	0.190	−0.625 to 0.924	0.304	0.299	−0.126 to 0.186	0.706
BMI	−0.416	−0.837 to 0.006	0.053	−0.266	−0.688 to 0.155	0.214	−0.481	−0.936 to −0.141	0.159	−0.173	−0.447 to 0.108	0.214
FBG	0.281	−0.509 to 1.072	0.483	−0.155	−0.945 to 0.636	0.700	−0.243	−0.804 to 0.448	0.316	−0.337	−0.857 to 0.174	0.198
HbA1c	−0.443	−1.281 to 0.395	0.298	−0.227	−1.065 to 0.611	0.594	−0.157	−0.811 to 0.494	0.817	0.351	−0.194 to 0.892	0.206
eGFR	0.042	−0.059 to 0.143	0.416	0.015	−0.086 to 0.117	0.764	−0.111	−0.727 to 0.504	0.892	0.054	−0.604 to 0.771	0.871
uACR	−0.003	−0.007 to 0.001	0.112	−0.089	−0.308 to 0.124	0.128	−0.043	−0.101 to 0.024	0.206	−0.146	−0.416 to 0.124	0.288
TyG	−0.268	−0.013 to −0.524	0.012	−0.501	−0.446 to −0.596	0.023	−0.187	−0.059 to −0.327	0.041	−0.117	−0.232 to −0.048	0.016

HRV, heart rate variability; DM, diabetes mellitus; BMI, body mass index; FBG, fasting blood glucose; HbA1c, glycated hemoglobin; eGFR, estimated glomerular filtration rate;uUACR, urinary albumin-creatinine ratio; TyG, triglyceride-glucose index; SDNN, standard deviation of NN intervals; RMSSD, root mean square of successive differences between adjacent N-N intervals; HF, high-frequency; TP, total power.

**Table 5:** Multiple logistic regression analysis of the associations between clinical parameters and risk of CAN.

Variables		CAN	
	OR	95% CI	*P* value
Gender	0.680	0.256 to 1.757	0.427
Age	1.012	0.971 to 1.056	0.562
DM duration	1.033	0.961 to 1.115	0.382
BMI	0.947	0.842 to 1.057	0.337
FBG	0.574	0.422 to 0.747	0.001
HbA1c	1.060	0.819 to 1.375	0.654
eGFR	1.016	0.985 to 1.047	0.311
uACR	1.010	1.001 to 1.023	0.117
TyG	1.811	1.660 to 1.898	0.001

CAN, cardiac autonomic neuropathy; DM, diabetes mellitus; BMI, body mass index; FBG, fasting blood glucose; HbA1c, glycated hemoglobin; eGFR, estimated glomerular filtration rate; uACR, urinary albumin-creatinine ratio; TyG, triglyceride-glucose index.

## 4 Discussion

This cross-sectional study investigated the association between the TyG index and CAN in T2DM cases. The present study provides a novel contribution by revealing a significant elevation of the TyG index in patients with CAN relative to non-CAN controls, suggesting a meaningful link between the product of FBG and TG and the presence of CAN. Notably, among the assessed blood parameters, only the TyG index demonstrated consistent and significant correlations with multiple established CAN measures, which are known predictors of adverse diabetic outcomes (18–20). After adjusting for key covariates, including age, sex, DM duration, HbA1c, FBG, uACR, and eGFR，participants with higher TyG levels exhibited significantly reduced SDNN and RMSSD, which primarily represent parasympathetic activity, as well as markedly lower HF and TP, which indicate overall autonomic modulation involving both sympathetic and parasympathetic pathways. Consistent with these findings, participants with defined CAN exhibited significantly higher TyG levels than those without CAN, even after multivariable adjustment.

CAN is a common and progressive microvascular complication of diabetes, characterized by an imbalance between sympathetic and parasympathetic activities, ultimately leading to dysfunctional cardiovascular autonomic nervous system. In the early stages, CAN is typically asymptomatic, and symptom onset often signifies advanced disease and is usually nonspecific (21). Prevalence estimates vary by diabetes type, ranging from 29% to 54% in T1DM and from 12% to 73% in T2DM (10, 22–24). Additionally, the duration of diabetes is also a key determinant of CAN incidence. CAN affects approximately 40% of T1DM cases with a disease course of over 25 years (23), and up to 60% of those with T2DM after 10–15 years (24). Notably, high rates of CAN are also found in individuals with newly diagnosed T2DM and in youth with either T1DM or T2DM (25–27). In line with prior findings, this study further demonstrated a CAN prevalence of 61.3% in the current cohort, with affected patients being of advanced age and having a longer disease course. The wide variation in prevalence reported in current studies is commonly observed and can be attributed to differences in study populations, diabetes types, and assessment methodologies. Furthermore, CAN is increasingly recognized as an independent predictor of a spectrum of serious outcomes, including diabetes progression, micro- and macro-vascular complications of diabetes, and major cardiovascular events such as heart failure and mortality (4, 7, 8, 28, 29). Our data indicated that patients in the CAN group were significantly more likely to have a prior history of cardiovascular disease, along with neuropathy and nephropathy. These findings carry considerable prognostic implications.

The pathogenesis and progression of CAN in diabetes are driven by multifactorial mechanisms that are not fully elucidated. Dysglycemia and broader metabolic derangements are central precipitating factors in the development of CAN. The underlying pathophysiology, however, depends on diabetes type. In T1DM, hyperglycemia and its downstream cellular mechanisms are predominant drivers. Meanwhile, in T2DM, IR and the components of metabolic syndrome interact in a more complex manner to promote CAN (14). IR is an integral pathway that drives the development and progression of diabetic complications, including CAN (30–34). The TyG index, calculated as ln[FBG (mg/dL) × TG (mg/dL)/2], serves as a reliable and widely accepted marker of IR (35). However, the value of the TyG index in CAN remains incompletely understood. This study aimed to better characterize its significance within our cohort. To comprehensively analyze the relationship between TyG and CAN, we employed a panel of cardiovascular autonomic indices, incorporating CART measures and HRV analysis. The diagnostic approach for CAN combines the CART for diagnosing the condition and HRV analysis for assessing sympathetic and parasympathetic activities, representing the gold standard methodology in contemporary clinical and research settings (36). Our novel findings demonstrate a singular association between the TyG index and CAN. Indeed, the TyG index was significantly elevated in participants with CAN compared with those without CAN at baseline. In addition, both CART measures and HRV indices differed significantly between the two groups. Furthermore, using Spearman's rank correlation to assess relationships between clinical parameters and CAN measures, we found that CAN severity was inversely correlated with multiple HRV indices, including SDNN, RMSSD, pNN50, VLF, LF, HF, and TP. Of note, among the comprehensive indices evaluated, only the TyG index exhibited a significant inverse association with key HRV indices; meanwhile, it was positively correlated with multiple CART measures. Most importantly, the TyG index remained an independent predictor of CAN even after adjusting for key demographic and metabolic confounders. ROC curve analysis confirmed that the TyG index possesses satisfactory discriminatory ability for CAN diagnosis. Its diagnostic performance remained robust even after excluding patients with diabetic peripheral neuropathy and cardiovascular diseases. This stable predictive capability indicates that the TyG index serves as a credible biomarker for CAN risk screening, and its predictive potency is minimally affected by common diabetic comorbidities. While our findings align with previous reports (33, 34, 37), we extend existing evidence by conducting a more thorough investigation of multiple risk factors and a comprehensive evaluation of diverse assessment indicators for CAN.

The TyG index is increasingly considered a valid predictor for assessing the pathogenesis of diabetes and associated complications. In addition to its well-documented associations with T2DM and DN, the TyG index serves as a pivotal biomarker that connects IR to a wide array of cardio-cerebrovascular and metabolic complications (15–17, 38, 39). Individuals with T2DM exhibit markedly elevated IR, which correlates strongly with uncontrolled glycemia that requires insulin therapy, as well as adverse outcomes such as DN and cardiovascular events (39, 40). Its links to atherogenic lipoprotein abnormalities and diabetes risk are well-documented, with longitudinal studies showing that changes in the TyG index correlate with altered diabetes incidence and risk (41). In the present study, the TyG index was consistently and significantly higher in the CAN group than in the non-CAN group. Previous data indicated that obese patients with T2DM exhibit suboptimal glycemic control and elevated TG levels, and are at elevated risk for developing CAN (33). The prevalence of hypertriglyceridemia has risen substantially and emerged as a major public health concern, paralleling the growing populations affected by obesity, metabolic syndrome, and T2DM. In this context, the TyG index represents a low-cost, readily accessible marker that may be more suitable for identifying and assessing diabetes-related metabolic dysregulation, including CAN. Future studies are warranted to elucidate the pathogenic mechanisms linking the TyG index to CAN and to assess its potential as a therapeutic target.

With the continuously escalating global prevalence of T2DM, the burden of CAN is expected to increase substantially among diabetic populations over the upcoming decades. Although the present study provides novel evidence supporting the clinical utility of the TyG index for CAN risk stratification, several noteworthy limitations should be acknowledged when interpreting the findings. First, the cross-sectional nature of this study precludes the establishment of causality between elevated TyG index and the development or progression of CAN. Further longitudinal and prospective investigations are therefore required to clarify the temporal sequence and underlying mechanistic pathways. Second, all participants were enrolled from a single tertiary hospital and exclusively consisted of inpatients, which may introduce inherent selection bias. Hospitalized patients typically exhibit more severe metabolic derangements and higher complication burdens, which may overestimate the overall CAN prevalence and the predictive magnitude of the TyG index, thereby limiting the generalizability to community-based or outpatient diabetic populations. Third, the CART grading system adopted in this study slightly differs from the conventional Ewing's criteria. Although this optimized grading strategy enables finer and more quantitative stratification of CAN severity, its relatively lenient threshold may potentially overdiagnose early-stage subclinical CAN cases. Fourth, this study lacked direct evaluations of sympathetic-parasympathetic balance, such as HRV-derived LF/HF ratio dynamics, as well as circulating catecholamine levels and their metabolites. The absence of these objective autonomic biomarkers restricts further exploration of the neurohumoral mechanisms linking insulin resistance to autonomic dysfunction. Future studies should integrate multiple glycemic biomarkers and dynamic autonomic parameters across diverse populations to further validate and optimize the predictive model. The final limitation of this study lies in its modest sample size of enrolled participants. Stratified subgroup analysis would split the full cohort into multiple underpowered subgroups, potentially generating unstable statistical results, amplified random errors, and biased correlation estimates. Consequently, stratified analyses were not carried out in the present study. In addition, classifying participants into three subgroups using dual cutoff values would further reduce the sample size of each subgroup, compromising the robustness and reliability of diagnostic parameter estimates for the two thresholds. Collectively, these limitations suggest that our results should be interpreted prudently, and larger-sample, multicenter, prospective studies with broader population coverage are warranted to validate and generalize our conclusions.

## 5 Conclusions

The TyG index was consistently associated with several standardized CAN measures in individuals with T2DM. The current results revealed a significantly higher TyG index in patients with CAN. As a well-established, effective, and practical surrogate marker of IR, this finding underscores the clinical relevance of the TyG index in diabetes-related complications. Further research is required to elucidate the mechanisms underlying the TyG-CAN association and to evaluate its potential for developing biomarkers to diagnose and monitor CAN progression.

## Data Availability

The original contributions presented in the study are included in the article/Supplementary Material, further inquiries can be directed to the corresponding authors.

## References

[1] Hubacek JA Dlouha L Adamkova V Dlouha D Pacal L Kankova K. Genetic risk score is associated with T2DM and diabetes complications risks. Gene. (2023) 849:146921. 10.1016/j.gene.2022.146921 36174902

[2] Genitsaridi I Salpea P Salim A Sajjadi SF Tomic D James S. 11th edition of the IDF Diabetes Atlas: global, regional, and national diabetes prevalence estimates for 2024 and projections for 2050. Lancet Diabetes Endocrinol. (2026) 14:149–56. 10.1016/S2213-8587(25)00299-2 41412135

[3] Li Y Teng D Shi X Qin G Qin Y Quan H. Prevalence of diabetes recorded in mainland China using 2018 diagnostic criteria from the American diabetes association: national cross sectional study. BMJ (Clinical Research ed). (2020) 369:m997. 10.1136/bmj.m997 32345662 PMC7186854

[4] Ang L Dillon B Mizokami-Stout K Pop-Busui R. Cardiovascular autonomic neuropathy: a silent killer with long reach. Auton Neurosci. (2020) 225:102646. 10.1016/j.autneu.2020.102646 32106052

[5] Ang L Mizokami-Stout K Eid SA Elafros M Callaghan B Feldman EL. The conundrum of diabetic neuropathies-past, present, and future. J Diabetes Complicat. (2022) 36:108334. 10.1016/j.jdiacomp.2022.108334 PMC10202025 36306721

[6] Zoppini G Cacciatori V Raimondo D Gemma M Trombetta M Dauriz M. Prevalence of cardiovascular autonomic neuropathy in a cohort of patients with newly diagnosed type 2 diabetes: the verona newly diagnosed type 2 diabetes study (VNDS). Diabetes Care. (2015) 38:1487–93. 10.2337/dc15-0081 26068862

[7] Williams S Raheim SA Khan MI Rubab U Kanagala P Zhao SS. Cardiac autonomic neuropathy in type 1 and 2 diabetes: epidemiology, pathophysiology, and management. Clin Ther. (2022) 44:1394–416. 10.1016/j.clinthera.2022.09.002 36272822

[8] Chowdhury M Nevitt S Eleftheriadou A Kanagala P Esa H Cuthbertson DJ. Cardiac autonomic neuropathy and risk of cardiovascular disease and mortality in type 1 and type 2 diabetes: a meta-analysis. BMJ Open Diabetes Res Care. (2021) 9:e2480. 10.1136/bmjdrc-2021-002480 PMC8719152 34969689

[9] Eleftheriadou A Williams S Nevitt S Brown E Roylance R Wilding JPH. The prevalence of cardiac autonomic neuropathy in prediabetes: a systematic review. Diabetologia. (2021) 64:288–303. 10.1007/s00125-020-05316-z 33164108 PMC7801295

[10] Ziegler D Voss A Rathmann W Strom A Perz S Roden M. Increased prevalence of cardiac autonomic dysfunction at different degrees of glucose intolerance in the general population: the KORA S4 survey. Diabetologia. (2015) 58:1118–28. 10.1007/s00125-015-3534-7 25724570

[11] Edwards JL Vincent AM Cheng HT Feldman EL. Diabetic neuropathy: mechanisms to management. Pharmacol Ther. (2008) 120:1–34. 10.1016/j.pharmthera.2008.05.005 18616962 PMC4007052

[12] Pang L Lian X Liu H Zhang Y Li Q Cai Y. Understanding diabetic neuropathy: focus on oxidative stress. Oxid Med Cell Longevity. (2020) 2020:9524635. 10.1155/2020/9524635 PMC7422494 32832011

[13] Achmad C Lim NS Pramudyo M Iqbal M Karwiky G Febrianora M. Relation between glycemic control and cardiac autonomic neuropathy in patients with diabetes mellitus type 2. Curr Probl Cardiol. (2023) 48:101135. 10.1016/j.cpcardiol.2022.101135 35124077

[14] Williams SM Eleftheriadou A Alam U Cuthbertson DJ Wilding JPH. Cardiac autonomic neuropathy in obesity, the metabolic syndrome and prediabetes: a narrative review. Diabetes Ther. (2019) 10:1995–2021. 10.1007/s13300-019-00693-0 31552598 PMC6848658

[15] Campos Muñiz C León-García PE Serrato Diaz A Hernández-Pérez E. Diabetes mellitus prediction based on the triglyceride and glucose index. Med Clin (Barc). (2023) 160:231–6. 10.1016/j.medcli.2022.07.003 35933191

[16] Su W Chen S Huang Y Huang JC Wu PY Hsu WH. Comparison of the effects of fasting glucose, hemoglobin A(1c), and triglyceride-glucose Index on cardiovascular events in type 2 diabetes Mellitus. Nutrients. (2019) 11:2838. 10.3390/nu11112838 31752391 PMC6893677

[17] Liu L Xia R Song X Zhang B He W Zhou X. Association between the triglyceride-glucose index and diabetic nephropathy in patients with type 2 diabetes: a cross-sectional study. J Diabetes Investig. (2021) 12:557–65. 10.1111/jdi.13371 33319507 PMC8015837

[18] Yan H Zhou Q Wang Y Tu Y Zhao Y Yu J. Associations between cardiometabolic indices and the risk of diabetic kidney disease in patients with type 2 diabetes. Cardiovasc Diabetol. (2024) 23:142. 10.1186/s12933-024-02228-9 38664793 PMC11046854

[19] Çatak M Konuk ŞG Hepsen S. The cholesterol-HDL-glucose (CHG) index and traditional adiposity markers in predicting diabetic retinopathy and nephropathy. J Diabetes Investig. (2025) 16:1487–94. 10.1111/jdi.70086 PMC12315246 40434226

[20] Zhang H Wang L Zhang Q Song Y Cai M Bao J. Non-linear association of triglyceride-glucose index with cardiovascular and all-cause mortality in T2DM patients with diabetic kidney disease: NHANES 2001−2018 retrospective cohort study. Lipids Health Dis. (2024) 23:253. 10.1186/s12944-024-02249-z 39154178 PMC11330591

[21] Spallone V. Update on the impact, diagnosis and management of cardiovascular autonomic neuropathy in diabetes: what is defined, what is new, and what is unmet. Diabetes Metab J. (2019) 43:3–30. 10.4093/dmj.2018.0259 30793549 PMC6387879

[22] Davis TME Tan E Davis WA. Prevalence and prognostic significance of cardiac autonomic neuropathy in community-based people with type 2 diabetes: the fremantle diabetes study phase II. Cardiovasc Diabetol. (2024) 23:102. 10.1186/s12933-024-02185-3 38500197 PMC10949593

[23] Pop-Busui R Braffett BH Zinman B Martin C White NH Herman WH. Cardiovascular autonomic neuropathy and cardiovascular outcomes in the diabetes control and complications trial/epidemiology of diabetes interventions and complications (DCCT/EDIC) study. Diabetes Care. (2017) 40:94–100. 10.2337/dc16-1397 27803120 PMC5180458

[24] Low PA Benrud-Larson LM Sletten DM Opfer-Gehrking TL Weigand SD O’Brien PC. Autonomic symptoms and diabetic neuropathy: a population-based study. Diabetes Care. (2004) 27:2942–7. 10.2337/diacare.27.12.2942 15562211

[25] Andersen ST Witte DR Dalsgaard E Andersen H Nawroth P Fleming T. Risk factors for incident diabetic polyneuropathy in a cohort with screen-detected type 2 diabetes followed for 13 years: ADDITION-Denmark. Diabetes Care. (2018) 41:1068–75. 10.2337/dc17-2062 29487078

[26] Mather KJ Bebu I Baker C Cohen RM Crandall JP DeSouza C. Prevalence of microvascular and macrovascular disease in the glycemia reduction approaches in diabetes—a comparative effectiveness (GRADE) study cohort. Diabetes Res Clin Pract. (2020) 165:108235. 10.1016/j.diabres.2020.108235 32450102 PMC7416515

[27] Jaiswal M Divers J Urbina EM Dabelea D Bell RA Pettitt DJ. Cardiovascular autonomic neuropathy in adolescents and young adults with type 1 and type 2 diabetes: the SEARCH for diabetes in youth cohort study. Pediatr Diabetes. (2018) 19:680–9. 10.1111/pedi.12633 29292558 PMC5938122

[28] Pop-Busui R Januzzi JL Bruemmer D Butalia S Green JB Horton WB. Heart failure: an underappreciated complication of diabetes. A consensus report of the American diabetes association. Diabetes Care. (2022) 45:1670–90. 10.2337/dci22-0014 35796765 PMC9726978

[29] Astrup AS Tarnow L Rossing P Hansen BV Hilsted J Parving HH. Cardiac autonomic neuropathy predicts cardiovascular morbidity and mortality in type 1 diabetic patients with diabetic nephropathy. Diabetes Care. (2006) 29:334–9. 10.2337/diacare.29.02.06.dc05-1242 16443883

[30] Dyck PJ Davies JL Litchy WJ O'Brien PC. Longitudinal assessment of diabetic polyneuropathy using a composite score in the Rochester diabetic neuropathy study cohort. Neurology. (1997) 49:229–39. 10.1212/WNL.49.1.229 9222195

[31] Toth C Brussee V Zochodne DW. Remote neurotrophic support of epidermal nerve fibres in experimental diabetes. Diabetologia. (2006) 49:1081–8. 10.1007/s00125-006-0169-8 16528572

[32] Yousri NA Suhre K Yassin E Al-Shakaki A Robay A Elshafei M. Metabolic and metabo-clinical signatures of type 2 diabetes, obesity, retinopathy, and dyslipidemia. Diabetes. (2022) 71:184–205. 10.2337/db21-0490 34732537 PMC8914294

[33] Akbar M Bhandari U Habib A Ahmad R. Potential association of triglyceride glucose Index with cardiac autonomic neuropathy in type 2 diabetes Mellitus patients. J Korean Med Sci. (2017) 32:1131–8. 10.3346/jkms.2017.32.7.1131 28581270 PMC5461317

[34] Jeyaseeli ARG Mathivanan D Prabagaran A. Assessment of triglyceride glucose index in type 2 diabetes mellitus patients with and without cardiac autonomic neuropathy. Cureus. (2023) 15:e42541. 10.7759/cureus.42541 37533622 PMC10393284

[35] Ramdas Nayak VK Satheesh P Shenoy MT Kalra S. Triglyceride glucose (TyG) Index: a surrogate biomarker of insulin resistance. J Pak Med Assoc. (2022) 72:986–8. 10.47391/JPMA.22-63 35713073

[36] Assessment: clinical autonomic testing report of the therapeutics and technology assessment subcommittee of the American academy of neurology. Neurology. (1996) 46:873–80. 8618715

[37] Huang Q Nan W He B Xing Z Peng Z. Association of baseline and trajectory of triglyceride-glucose index with the incidence of cardiovascular autonomic neuropathy in type 2 diabetes mellitus. Cardiovasc Diabetol. (2025) 24:66. 10.1186/s12933-025-02622-x 39920656 PMC11806751

[38] Abbasi F Reaven GM. Comparison of two methods using plasma triglyceride concentration as a surrogate estimate of insulin action in nondiabetic subjects: triglycerides×glucose versus triglyceride/high-density lipoprotein cholesterol. Metabolism. (2011) 60:1673–6. 10.1016/j.metabol.2011.04.006 21632070

[39] Qiu J Li J Xu S Zeng H Zhang Y Yang S. Can cardiovascular health and its modifiable healthy lifestyle offset the increased risk of all-cause and cardiovascular deaths associated with insulin resistance? Cardiovasc Diabetol. (2025) 24:114. 10.1186/s12933-025-02674-z 40065337 PMC11895255

[40] Parwani K Mandal P. Advanced glycation end products and insulin resistance in diabetic nephropathy. Vitam Horm. (2024) 125:117–48. 10.1016/bs.vh.2024.02.007 38997162

[41] Lee S Yang HK Ha H Lee J Kwon H Park Y. Changes in metabolic health Status over time and risk of developing type 2 diabetes: a prospective cohort study. Medicine (Baltimore). (2015) 94:e1705. 10.1097/MD.0000000000001705 26448024 PMC4616763

